# Differences in Genotype and Virulence among Four Multidrug-Resistant *Streptococcus pneumoniae* Isolates Belonging to the PMEN1 Clone

**DOI:** 10.1371/journal.pone.0028850

**Published:** 2011-12-19

**Authors:** N. Luisa Hiller, Rory A. Eutsey, Evan Powell, Joshua P. Earl, Benjamin Janto, Darren P. Martin, Suzanne Dawid, Azad Ahmed, Mark J. Longwell, Margaret E. Dahlgren, Suzanne Ezzo, Herve Tettelin, Sean C. Daugherty, Timothy J. Mitchell, Todd A. Hillman, Farrel J. Buchinsky, Alexander Tomasz, Herminia de Lencastre, Raquel Sá-Leão, J. Christopher Post, Fen Z. Hu, Garth D. Ehrlich

**Affiliations:** 1 Allegheny General Hospital, Allegheny-Singer Research Institute, Center for Genomic Sciences, Pittsburgh, Pennsylvania, United States of America; 2 Institute of Infectious Disease and Molecular Medicine, University of Cape Town, Cape Town, South Africa; 3 Department of Pediatrics and Communicable Diseases, University of Michigan, Ann Arbor, Michigan, United States of America; 4 Institute for Genome Sciences, Department of Microbiology and Immunology, University of Maryland School of Medicine, Baltimore, Maryland, United States of America; 5 Institute of Infection, Immunology and Inflammation, University of Glasgow, Glasgow, Scotland; 6 Department of Microbiology and Immunology, Drexel University College of Medicine, Allegheny Campus, Pittsburgh, Pennsylvania, United States of America; 7 Department of Otolaryngology/Head and Neck Surgery, Drexel University College of Medicine, Allegheny Campus, Pittsburgh, Pennsylvania, United States of America; 8 Rockefeller University, New York, New York, United States of America; 9 Instituto de Tecnologia Química e Biológica, Universidade Nova de Lisboa, Oeiras, Portugal; The University of Hong Kong, Hong Kong

## Abstract

We report on the comparative genomics and characterization of the virulence phenotypes of four *S. pneumoniae* strains that belong to the multidrug resistant clone PMEN1 (Spain^23F^ ST81). Strains SV35-T23 and SV36-T3 were recovered in 1996 from the nasopharynx of patients at an AIDS hospice in New York. Strain SV36-T3 expressed capsule type 3 which is unusual for this clone and represents the product of an *in vivo* capsular switch event. A third PMEN1 isolate – PN4595-T23 – was recovered in 1996 from the nasopharynx of a child attending day care in Portugal, and a fourth strain – ATCC700669 – was originally isolated from a patient with pneumococcal disease in Spain in 1984. We compared the genomes among four PMEN1 strains and 47 previously sequenced pneumococcal isolates for gene possession differences and allelic variations within core genes. In contrast to the 47 strains – representing a variety of clonal types – the four PMEN1 strains grouped closely together, demonstrating high genomic conservation within this lineage relative to the rest of the species. In the four PMEN1 strains allelic and gene possession differences were clustered into 18 genomic regions including the capsule, the blp bacteriocins, erythromycin resistance, the MM1-2008 prophage and multiple cell wall anchored proteins. In spite of their genomic similarity, the high resolution chinchilla model was able to detect variations in virulence properties of the PMEN1 strains highlighting how small genic or allelic variation can lead to significant changes in pathogenicity and making this set of strains ideal for the identification of novel virulence determinants.

## Introduction

The gram-positive bacterium *Streptococcus pneumoniae* (commonly referred to as pneumococcus) is a major human pathogen. While pneumococcal disease is common, asymptomatic colonization is many times more prevalent, especially among young children [1], [2]. Variation in the pathogenic potential of individual strains often correlates with substantial differences in the gene complements displayed by clinical isolates [3]–[5]. The whole genome sequence (WGS) is now available for many pneumococcal stains, and on average, pairs of isolates have hundreds of gene possession differences [6]–[15]. This variability is attributed to frequent horizontal gene transfer (HGT) events from both pneumococci and related species [11], [16], [17].

The three major penicillin resistant (and multidrug resistant) clones of *S. pneumoniae* – PMEN1 (Spain^23F^ ST81), PMEN2 (Spain^6B^ ST90) and PMEN3 (Spain^9V^ ST156) – stand out in sharp contrast to the tremendous genetic variability of the pneumococci as a whole since each one of these drug-resistant clades displays decreased genomic diversity when compared to the rest of the species [18]. The PMEN1 clone is estimated to have originated around 1970, and is widely distributed throughout Europe, Asia, Africa and the Americas [9]. PMEN1 isolates are multilocus sequence type (MLST) 81, have a common pulse field gel electrophoresis profile (PFGE), possess consistent multilocus enzyme electrophoresis (MEE) patterns, and have identical penicillin binding protein (PBP) patterns and ribotypes [19]. Besides penicillin resistance, most PMEN1 isolates are also resistant to chloramphenicol and tetracycline, and many isolates have additional resistance to fluoroquinolones and macrolides [20], [21]. Although predominantly of serotype 23F, some isolates have switched their capsular type to 9N, 19A, 19F, 14, 6A, 15B, and 3 [9], [19]. In addition to causing serious disease, isolates belonging to the PMEN1 clone are also frequent colonizers [22].

We hypothesized that PMEN1 strains vary in their capacity to cause otoscopic and systemic disease, and contain genic differences that are much more extensive than the variability in capsule and antibiotic-resistance observed by molecular typing techniques. Thus, we compared the whole genome sequence (WGS) of four nasopharyngeal PMEN1 isolates, and investigated their capacity to cause disease in the chinchilla model of otitis media.

## Materials and Methods

### Strains and DNA sequencing

Strain ATCC700669, which is serotype 23, was obtained from Dr. Mitchell, it was isolated from the nasopharynx of a patient in 1984 in Spain, and was selected as a representative of the original penicillin-resistant clone linked to the Spanish PMEN1 epidemic of the 1980s. Strains SV35-T23 and SV36-T3 were recovered from the nasopharynx of patients attending the AIDS clinic of the St. Vincent's Medical Center in Richmond, New York in 1996 [19]. One of these isolates – SV36-T3 – expresses capsule type 3 instead of the more common type 23F and seems to represent the product of an *in vivo* capsular switching event [19]. Both of these strains were recovered at the same hospital and the same time, and were compared to determine whether they differed only by a capsule switch (which would suggest that they represent a parental and recombinant pair) or whether more extensive differences would be detected. Strain PN4595-T23 was recovered from the nasopharynx of a healthy child at a day-care center in Lisbon, Portugal in 1996 [23]. The comparison of this carriage strain to the other strains serves to investigate one example of how geographic location or date of isolation may correlate with strain variability. Strain SV35-T23SV36blp is a derivative of strain SV35-T23 constructed in the laboratory to carry the blp bacteriocin locus from SV36-T3. The genome of strain ATCC700669 is published (8). The genomes of strains SV35-T23, SV36-T3, and PN4595-T23 were sequenced at the Center for Genomic Sciences (CGS) using a 454 Life Sciences FLX sequencer. Strains were sequenced to a depth of 21-fold or greater and were assembled by the 454 Newbler *de novo* assembler. All strains were grown in Todd-Hewitt broth without aeration to delay exponential phase of growth.

### Genome assembly of CGS *S. pneumoniae* sequences

The 454-assembled genomic contigs were ordered and oriented into scaffolds by alignment, using the computer program, Nucmer [24], against the WGS of *S. pneumoniae* strain ATCC700669 [8]. Using a maximum-parsimony approach, genomes SV35-T23 and SV36-T3 were reduced to about 12 contigs by a combination of: (i) Sanger sequencing of PCR amplicons targeted to fill gaps between neighboring contigs, as inferred from the scaffolding, and (ii) paired-end Sanger sequencing of clones from a library and identification of clones that spanned gaps in the 454 sequence. Gap closure experiments were designed by a custom Perl script, and PCR primers were designed by Primer3 [25]. Clones and PCR amplicons were assembled along with 454 contigs by a modified Phred-Phrap-Consed pipeline where 454 contigs were converted to PHD format files and were input into Phrap as long reads [26]. Data were manipulated and visualized using Consed [27]. We have deposited the contig data at GenBank under accession numbers, ADNN, ADNO, ABXO for SV35-T23, SV36-T3, and PN4595-T23 respectively.

### Gene prediction

Using Mauve Genome Alignment Software, the final contigs were assembled *in silico* into one linear scaffold based on the complete sequence of ATCC700669 (GenBank FM211187). The mapping of the sequenced contigs onto the final assembly is displayed in Table S1. Using the Strepneumo database (http://strepneumo-sybil.igs.umaryland.edu/), from Integrated Genome Science (IGS), this continuous scaffold was used for prediction of putative coding sequences and gene annotations.

### Clustering algorithm

A complete description of the algorithms used to create the orthologous clusters is given by Hogg et al. [28]. Briefly, to allocate the genes into core or distributed subsets, tfasty34 (Fasta package, version 3.4) was used for six-frame translation homology searches of all predicted proteins against all possible translations. Software designed at the CGS was used to parse this output, grouping the genes into clusters. A cluster was defined as a group of genes within which each individual (1) shares at least 70% identity, over 70% of their lengths with one or more of the other genes in the group, and (2) where at least one sequence in the cluster is longer than 119 residues.

### Graph of Strain Difference

For the genic and allelic difference-based graphs, 51 *S. pneumoniae* strains (WGS) were compared. In addition to the three newly sequenced PMEN1 strains presented here, the other 48 strains have been previously published. Forty-four of these genomes (including the ATCC700669) are described by Donati et al., and correspond to a highly diverse set of strains (spanning 19 serotypes, 24 MLST CCs, and including laboratory, disease, and carriage-associated strains from multiple geographic locations) [11]. The remaining four strains (ST13v1:ABWQ; ST2011v4:ADHN; ST13v6:ABWB; and ST13v12:ABWU) were isolated from one patient and are described in Hiller et al. [29]. Genic distance measures between genomes were identified as the total commonality between each pairwise subset's distributed genes divided by the total number of 1607 distributed genes. Commonality included the case where both genomes either contained the distributed gene, or did not contain a given distributed gene that was present in some number of other strains. The normalized commonality score was then subtracted by one to give a distance metric between each pair of genomes [30]. Allelic distance measures between genomes are directly proportional to the percent identity among all the 1324 core alleles. The distance metrics were used to create a neighbor joining tree using the computer program NEIGHBOR in the PHYLIP package (Version 3.69) [31]. The computer program Figtree (Version 1.3.1) was used to visualize the tree using a midpoint root (freely available from http://tree.bio.ed.ac.uk/software/figtree/).

### Phylogenetic Tree

A plylogenetic tree for the four genotypes was constructed using the alignment generated by Mauve [32] and the neighbor joining algorithm implemented in RDP3 [33].

### Single nucleotide polymorphism and insertion/deletion predictions

SNPs from the WGS were identified using the tab-delimited SNP file produced by Mauve 2.3 [32].

### Recombination analysis

To detect PMEN1 genomic sites that arose by recombination events, we used Mauve to align multiple genomes, and RDP3 for recombination detection [33], [34]. This analysis included the four PMEN1 sequences, and an additional seven pneumococcal full genome sequences, selected from the set of 48 genomes described above, based on their highly variable genomic content. The seven genomes are: AP200, CDC0288-04, CDC3059-06, D39, TIGR4, JJA, and CGSSp6BS73, and their respective serotype/MLST types are: 11A/62; 12F/220; 19A/199; 2/595; 4/205; 14/66; and 6/460. For RDP3, default settings were selected, except that RDP, MAXCHI, CHIMAERA, BOOTSCAN and SISCAN window settings were changed to 50, 120, 100, 2000 and 2000 nucleotides respectively and only recombination signals detectable with four or more of the seven recombination detection methods implemented in RDP3 were considered as sufficient evidence of recombination. RDP3 identifies recombinant sequences and possible recombination breakpoint positions within these.

### Construction of recombinant SV35-T23SV36blp strain

The construction of SV35-T23-SV36blp was a two-step process. The first consisted of inserting a resistance cassette upstream of the bacteriocin locus in SV36-T3. For this, 2 Kb flanks were PCR amplified on each side of the 282 bp space between genes spiA and blpI. The spiA flank contained the restriction site BamHI on the end of its reverse primer, while the blpI flank contained an XmaI site on the end of its forward primer. A spectinomycin resistance cassette was amplified from plasmid pR412 using primers with a BamHI site on the forward promoter and an XmaI site on the reverse primer. All these products were digested with their respective enzymes and ligated together to form a spiA flank-spectinomycin resistance cassette-blpI flank. This ligation product was amplified using internal primers, and the product was transformed into strain SV36-T3 by natural transformation, and resistance clones were confirmed by PCR. The second step involved switching the SV35-T23 bacteriocin and immunity protein regions with those of SV36-T3. To this end, the SV36-T3 spectinomycin clone was used as a template to PCR amplify the bacteriocins and immunity proteins in the blp region (9874 bp + resistance cassette). This PCR product was transformed into SV35-T23, and resistant clones were confirmed to be positive by PCR and Sanger sequencing. All primers used to create this construct are listed in Table S2.

### Construction of 4595-deletion mutant of PavB

The PavB gene was replaced by a spectinomycin resistance cassette. Using PCR and ligations, a linear construct was created that contained the 2 Kb upstream and the first 58 bp of PavB, a resistance cassette, and the 165 bp at the end of PavB plus another 2 Kb downstream. 5 µg of this linear construct was used for natural transformation into strain PN4595-T23. The absence of PavB in the resistant colonies was confirmed by PCR and Sanger sequencing. All primers used to create this construct are listed in Table S2.

### Genetic transformation of *S. pneumonia* strains in the laboratory

Bacterial cultures were grown to optical density of 0.05 in Columbia broth. Next, 1 ml of culture was mixed to 50 µl BSA (4%), 5 µl CaCl2 (1%), 5 µl of competence stimulating peptide (CSP) at 20 µg/ml, and 1–5 µg of DNA. For PMEN1 strains tCSP-2 was used. The mixture was incubated at 37°C for 2 hours and plated on selective media. Plates were incubated at 37°C overnight to allow bacterial growth.

### Colony morphology

Serotype 23F strains (ATCC700669, PN4595-T23, and SV35-T23) were plated on tryptic soy agar after growing in 5% CO_2_ overnight, and colony morphology was analyzed under a dissection microscope to distinguish between opaque and transparent colonies.

### Inhibition assay for intra-species competition

Agar overlay assays were preformed as previously described [35]. Briefly, overnight growth of the strain to be tested for inhibition was taken from tryptic soy agar (TSA) plates supplemented with 5% sheep blood and stabbed into a TSA plate supplemented with catalase. After 6 hours of growth at 37°C in 5% CO_2_, the plate was carefully overlayed with a mixture of TSA, 0.5% agar, catalase and 200 µl of a mid-log phase grown culture of the indicator strain P537. Growth was allowed to continue overnight and bacteriocin mediated inhibition was documented by observing a zone of inhibition around the stabbed strain.

### Phenotypic comparison in the chinchilla OM model

All chinchilla experiments were conducted with the approval of the Allegheny-Singer Research Institute (ASRI) Institutional Animal Care and Use Committee (IACUC), Animal Research Protocol #864. Cultures were grown in Todd-Hewitt broth and diluted in buffered physiological saline solution. Cohorts of at least ten young adult chinchillas (*Chinchilla laniger*) (McClenahan Chinchilla Ranch, New Wilmington, PA) were injected bilaterally with 100 µl into the tympanic bullae with 10^2^ CFU of the respective *S. pneumoniae* strains using a 27-gauge needle. Before inoculation and otoscopic reading, anesthesia was induced using a solution of ketamine hydrochloride 100 mg/mL, xylazine hydrochloride 30 mg/mL and acepromazine 5 mg/mL.

A multi-parameter scoring system based on otoscopic and systemic disease was applied daily for ten days to assess the degree of disease induced by each individual strain [4]. Otologic disease ranged from no disease with a score of 1 to a ruptured tympanic membrane with a score of 4, and systemic disease ranged from healthy animals with a score of 0 to a dead animal with a score of 4 (scale outlined in Table S3). Strains were compared for maximal otoscopic and systemic disease, rate of fluid development in the middle ear, rate at which symptoms of illness developed, and the number of animals that died or reached a maximal systemic disease score and were euthanized.

## Results

### Sequencing and assembly of PMEN1 *S. pneumoniae* isolates

A summary of the strain history, as well as sequencing and assembly details for four PMEN1 strains is presented in Table 1. The three newly sequenced genomes (SV35-T23, SV36-T3, and PN4595-T23) have a GC content of 39% and have an average size of 2.1 Mb. The Strepneumo database was used to identify and annotate the coding sequences for each of these genomes which averaged 2206 genes/genome (Table 2). Their complete genome sequences have been deposited in GenBank (ADNN for SV35-T23, ADNO for SV36-T3, and ABXO for PN4595-T23) and at the Strepneumo database http://strepneumo-sybil.igs.umaryland.edu/.

**Table 1:** Strain history, sequencing and assembly information on PMEN1 strains.

Strain	SV35-T23	SV36-T3	PN4595-T23	ATCC700669
**Isolation Date**	1996	1996	1996	1984
**Isolation Site**	hospital New York	hospital New York	day care Lisbon	hospital Barcelona
**Serotype**	23F	3	23F	23F
**MLST**	81	81	81	81
**Genbank ID**	ADNN	ADNO	ABXO	FM211187
**Sequencing Center**	Center for Genomic Sciences	Center for Genomic Sciences	Center for Genomic Sciences	Sanger
**CG%**	39.40	39.43	39.40	39.49
**Contig Length (bp)**	2133603	2114581	2152559	2221315
**Assembly Length (bp)**	2156885	2162633	2169192	2221315
**Read Coverage**	26	25.9	27.6	8
**Sequencing Platform**	FLX Standard	FLX Standard	FLX Standard	Applied Biosystems 3700
**Average Read Length**	260.3	260.89	250	N/A
**No reads assembled**	213488	210071	2155226	N/A
**Newbler Contigs**	89	94	96	N/A
**Final Contigs after closure**	12	10	47	1

**Table 2:** Summary of CDSs and their organization into homologous gene clusters.

Strain	CDSs	Total Gene Clusters	Distributed Gene Clusters	Core Gene Clusters
**SV35-T23**	2242	2075	28	2041
**SV36-T3**	2239	2064	16	2041
**PN4595-T23**	2259	2126	83	2041
**ATCC700669**	2084	2126	83	2041
**Total**	N/A	2142	94	2041

### Isolates of the PMEN1 lineage differ significantly in their ability to cause otologic and systemic disease in the chinchilla model of otitis media

**Figure 1:**
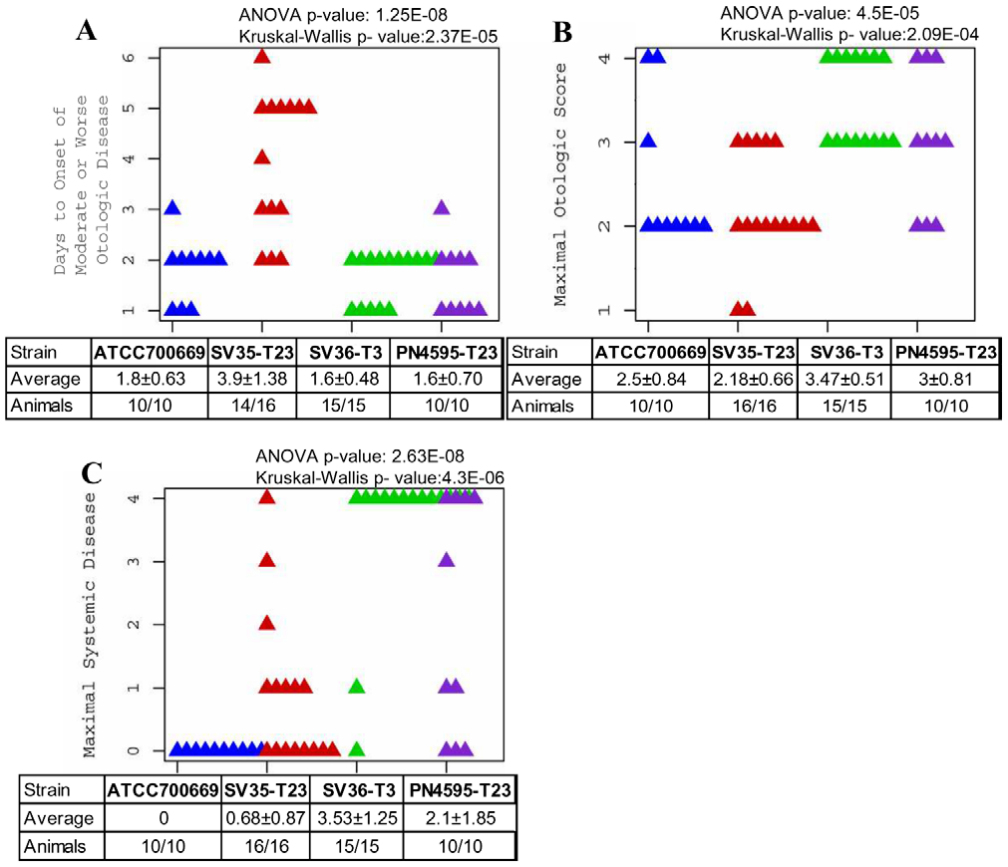
Extensive Phenotypic Variation among PMEN1 strains. Scatter plots illustrating differences in the outcome of otologic and systemic disease for animals infected with one of four PMEN1 strains. Parameters are: days to onset of moderate or worse otologic disease (A), maximal otologic disease (B), and maximal systemic disease (C).

The four PMEN1 isolates showed varying degrees of systemic and otologic disease in the chinchilla model of OM disease (Fig. 1). This model is ideal for virulence studies, since it is a reproducible model of local and systemic infection [4] and the extent of bacterial virulence in this model has been demonstrated to correlate with extent of disease in humans [36]. However, as the bacteria are injected directly into the middle ear, it does not measure nasopharyngeal colonization.

The rapidity of otologic disease was defined as the number of days to the appearance of unambiguous otological symptoms (minimal score of 2). All PMEN1 isolates induced otologic disease in the chinchilla model (Fig. 1A; note, that two animals infected with SV35-T23 were excluded from this set, as these specific animals never developed otologic disease). Analysis of the strain differences produced highly significant results using both ANOVA and the non-parametric Kruskal-Wallis test (p-value = 1.25×10^−8^ and 2.37×10^−5^, respectively). Analysis of all strain pairs using Tukey HSD demonstrated that SV35-T23 differed significantly from the other three strains in the onset of otologic disease. To measure the severity of local disease we used the maximal otologic score (Fig. 1B). Both ANOVA and Kruskal-Wallis tests showed significant differences among this set with p- values of 4.5×10^−5^ and 2.09×10^−4^, respectively. Furthermore, Tukey HSD comparisons of all strain pairs showed no statistical difference between SV36-T3 and PN4595-T23, but a statistical difference between these strains and SV35-T23, as well as between SV36-T3 and ATCC700669.

The four isolates also displayed differences in their capacity to cause systemic disease (Fig. 1C). Analysis of these strain differences were statistically significant using both ANOVA and the non-parametric Kruskal-Wallis test (p-value = 2.68×10^−8^ and 4.3×10^−6^, respectively). Furthermore, Tukey HSD analysis of the strain pairs shows at least three distinct phenotypes. The most virulent strain SV36-T3 differs significantly from ATCC700669, SV35-T23, and PN4595-T23 (p-values of 10^−7^, 2.4×10^−6^, and 0.036 respectively). The systemically avirulent strain, ATCC700669 (serotype 23), does not differ from SV35-T23, but does differ from PN4595-T23 (p-value = 0.0027). Together these results suggest at least three distinct systemic phenotypes of low, intermediate and high virulence. In agreement, differences in the moribundity of the chinchillas after infection with the individual strains were significant as determined by a Fisher's Exact test (p-value = 3.25×10^−7^) (Table 3).

**Table 3:** Strain Specific Moribundity and Spread of Infection to Lungs and Brain.

Strain	ATCC700669	SV35-T23	SV36-T3	PN4595-T23	Fisher-Exact p-value
Moribundity	0/10 - 0%	1/16 - 6%	13/15 - 86%	4/10 - 40%	3.25E-07
Bacteria in the Brain	0/10 - 0%	4/16 - 25%	11/15 - 73%	8/10 - 80%	4.98E-05
Bacteria in the Lung	1/10 -10%	3/16 - 19%	11/15 - 73%	3/10 - 30%	0.0026

Finally, we determined the number of animals where bacteria spread to the lung or brain to provide an overview of the mechanism that may have led to moribundity. Autopsies showed that some of the strains were much more likely to spread to the brain and/or lungs than others (Table 3). These differences are significant with p-values from the Fisher-Exact test of 4.98×10^−5^ for the brain and 2.6×10^−3^ for the lungs.

### PMEN1 strains form a highly conserved *S. pneumoniae* lineage, which nonetheless displays multiple differences among isolates

**Figure 2:**
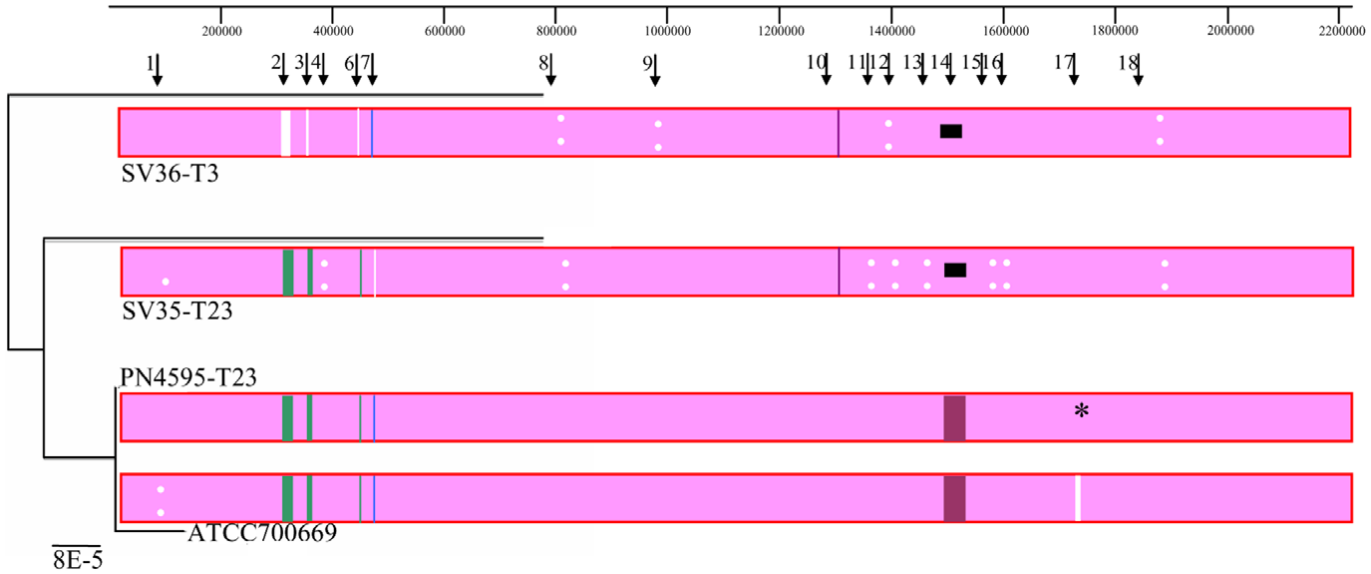
Recombination analyses of *Streptococcus pneumoniae* genomes. Schematic based on Mauve alignments of the whole genome sequences of four PMEN1 *S. pneumoniae* isolates. Colored blocks correspond to: pink, regions that are homologous in all four genomes and free from genomic rearrangement; green, regions that differ in strain SV36-T3; blue, regions that differ in strain SV35-T23; purple, regions that differ in strains SV35-T23 and SV36-T3; white, unique to one sequence. White dots highlight polymorphic regions. Asterisks denote the position of contig gaps. Horizontal black lines correspond to gaps within sequenced regions. This schematic does not indicate sequence gaps that are positioned at contig breaks (and thus likely to be the result of incomplete sequencing) or differences within ribosomal RNA encoding sequences (since these are often misassembled). Arrows at the top denote the position of the 18 NGs identified based on SNP strain differences. The tree on the left side corresponds to a neighbor joining phylogenetic tree expressing the relationship among the four PMEN1 isolates.

The WGS of SV35-T23, SV36-T3, PN4595-T23, and ATCC700669 were aligned using the progressive Mauve feature in the Mauve genome alignment software [32]. This alignment was used to create the schematic similarity plot displayed in Fig. 2. The genome sequences are displayed as collinear blocks and provide evidence of extensive synteny across all four genomes. The pink color observed over the majority of the alignment denotes regions conserved across all strains, demonstrating high similarity in this sequence set. The other colors display regions conserved among subsets of genomes, while white blocks show regions with genes unique to a single genome, and white dots denote regions with multiple single nucleotide polymorphisms (SNPs). To assess the relationships among these strains using the WGS a Neighbor Joining tree was created from the alignment as implemented in the Recombination Detection Program (RDP3) (left on Fig. 2). This tree reinforces the extremely high genomic similarity between ATCC700669 and PN4595-T23, as well as the genomic differences between this pair and the other two strains.

To create a quantitative map of all the nucleotide differences amongst the four genomes, all of the SNPs were identified. To eliminate any SNPs that were possibly due to sequencing artifacts, all base pairs with a quality score of less than 40 (a probability of >1∶10^4^ that they were incorrectly called) were eliminated from the analysis. Using the Mauve program, 3705 SNPs were identified. These SNPs were sorted based on their chromosomal placement, and subsequently manually curated to group together SNPs that are located within an area where there is a high concentration of SNPs (ranged from 2.6 to 233 SNPs/Kb) and where the majority of the SNPs shared the same distributions (that is, the strain(s) with the variable nucleotide was the same for the vast majority of the SNPs). Such groups are hereafter referred to as neighbor groups (NGs) [29], and are presented in Table 4 with their positions displayed in Fig. 2. Eighty per cent of the SNPs can be organized into 18 NGs, ranging from 0.04–46 Kb in size, and which fall into four strain distribution patterns.

**Table 4:** Summary of the genic and allelic differences among PMEN1 strains.

NG ID	Start Position on ATCC700669	End Position on ATCC700669	Size (bp)	No SNPs	No Genic Differences	Most Divergent Strain	Annotation Comments	CDSs within NG	RDP	RDP p-value
1	88645	89843	1198	140	0	ATCC700669	CWAP	SPN23F00940	**x**	2.30E-264
2	298906	331523	32617	855	19	SV36 -T3	Capsule	SPN23F03130-03400	**x**	0.00E-01
	342164	351416	9252	89		mixed		SPN23F03500-03600		
3	351416	364693	13277	164	7	SV36-T3	PTS	SPN23F03600-03770	**x**	6.00E-248
4	370726	396771	26045	151	0	SV35-T23		SPN23F03830-04130	**x**	8.95E-67
5	415590	419368	3778	30	0	SV35-T23	uncharacterized	SPN23F04320-04340		
6	445943	446512	569	133	0	SV36-T3	RM	SPN23F04590-04591		
7	458935	501248	42313	745	13	SV35-T23	blp	SPN23F04700-05210	**x**	E-308
8	791373	793728	2355	85	0	SVs	RM and phage proteins	SPN23F08090-08130		
9	985450	985498	48	10	0	SV36-T3	transposase	SPN23F10210		
10	1269364	1283683	14319	38	2	SVs	ery	SPN23F12950-13090		
11	1372062	1372560	498	31	0	SV35-T23	transposase	SPN23F14040		
12	1393131	1397181	4050	236	0	SVs		SPN23F14350-14390	**x**	2.7E-318
13	1453975	1462857	8882	42	0	SV35-T23	CWAP	SPN23F14980-15110	**x**	2.93E-26
14	1483547	1529527	45980	23	51	SVs	phage	SPN23F15250-15830		
15	1544508	1546773	2265	30	0	SV35-T23	Major facilitator	SPN23F16010-16030	**x**	2.93E-60
16	1575110	1599959	24849	231	0	SV35-T23	Membrane plus	SPN23F16360-16600	**x**	2.31E-231
17	1720188	1722292	2104	26	2	SVs	CWAP	SPN23F17820	**x**	1.43E-29
18	1831969	1839785	7816	31	0	SVs	CWAP	SPN23F18850-18960	**x**	8.43E-56
SUM			242215	3090	94					

Analysis of the four genomes with seven different recombination analysis methods implemented in the program RDP3 clearly indicated that at least 11 out of these 18 NGs were most likely attributable to homologous recombination between the PMEN1 isolates and other *S. pneumoniae* strains, or bacteria from other species (Figure S1). These regions of recombination include the capsule, a phosphotransferase system (PTS), a bacteriocin locus, and multiple cell wall associated proteins (CWAP). As might be expected the NGs that were not predicted to result from homologous recombination include the genes with an integrative and conjugative element (ICE) (NG10), a phage (NG14), transposases (NG9 and NG11), and restriction modification systems (NG6 and NG8).

Next, we performed a comprehensive analysis of the differences in gene possession among these strains. All coding sequences (CDS) from the four strains were grouped into homologous gene clusters as described in Hogg et al. [28] and then divided into core or distributed gene clusters. A distributed cluster was defined as any homologous gene cluster not present in all strains. These strains have a total of 2142 homologous clusters, of which only 94 are distributed (Table 2; detailed list and annotation in Table S4). All 94 distributed CDS are located within one of the 18 NGs. Thus, the major differences among these four strains exist within the 18 NG regions that collectively cover ∼240 Kb (12% of the genome) and that differ by >3000 SNPs and 94 genes (Table 4).

**Figure 3:**
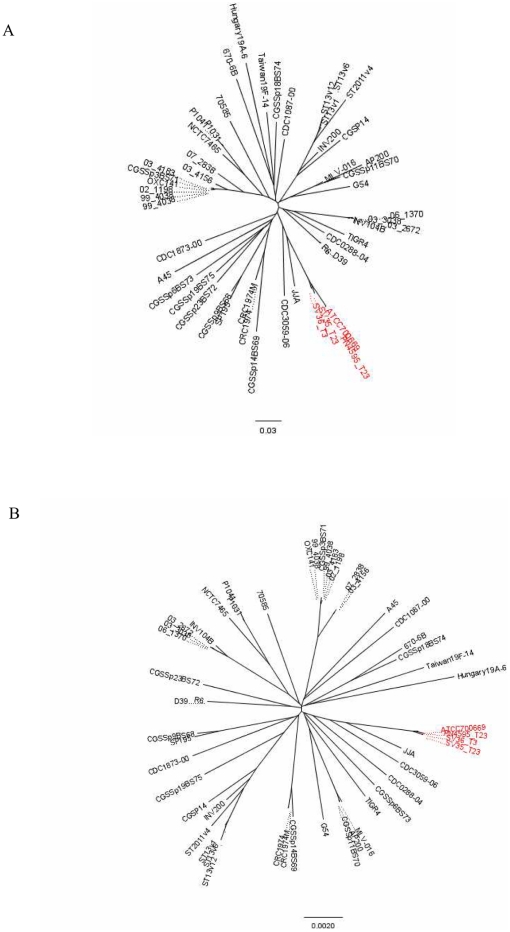
High degree of genomic similarity among PMEN1 strains. Grouping of 51 *S. pneumoniae* strains based on (**A**) the number of distributed genes or (**B**) the number of variable core alleles. These trees provide a measure of the genic or alleic distance between strains without making any inference regarding their phylogeny, since the high rates of recombination within the population can interfere with population-wide phylogenetic results. The PMEN1 genomes are more closely related to one-another than any one isolate is to any other independently isolated strains.

To place the differences among the PMEN1 strains in a species-wide perspective, we quantified the similarity and differences among these strains and another 48 previously published diverse pneumococcal isolates [11], [29], using both genic (distributed genes) and allelic differences (Fig. 3 A and B, respectively). Note that these diagrams do not include multiple isolates from other highly conserved lineages of pandemic multi-drug resistant strains since the whole genome sequences of such isolates are still unavailable. The distributed gene distance measured between genomes was defined as the number of distributed gene clusters shared or not shared by a given strain pair divided by the 1607 total distributed gene clusters found in the 51 strains [30]. The core gene distance measure was defined as the number of variable alleles within the core set of 1324 gene clusters. In both analyses the PMEN1 isolates are grouped closely together confirming the high similarity of their genomic content and demonstrating that this genome wide analysis is in agreement with a strain comparison using MLST genes. The result justifies their classification as a discrete lineage, even after the identification of multiple differing regions.

In summary, strains from the PMEN1 lineage group together relative to other pneumococcal lineages, but have many more differences than anticipated from the molecular typing (MLST, PFGE, MEE, PBP profiles) done within the clade, which shows few or no differences [19]. There are few differences between the phenotypically diverse strains ATCC700669 and PN4595-T23 (visually undistinguishable in the trees presented in Fig. 3A and B). Given that these strains were isolated 15 years apart, this demonstrates that PMEN1 isolates can persist with only minor *detectable* changes (homologous recombination between identical or nearly identical regions cannot be discounted). Nonetheless, the differences between the ATCC700669/PN4595-T23 pair, SV35-T23 and/or SV36-T3 demonstrate that PMEN1 isolates have accumulated multiple changes (beyond the capsular switch), as described in detail below.

### Detailed differences among PMEN1 isolates

#### Capsular loci and the downstream phosphotransferase system

**Figure 4:**
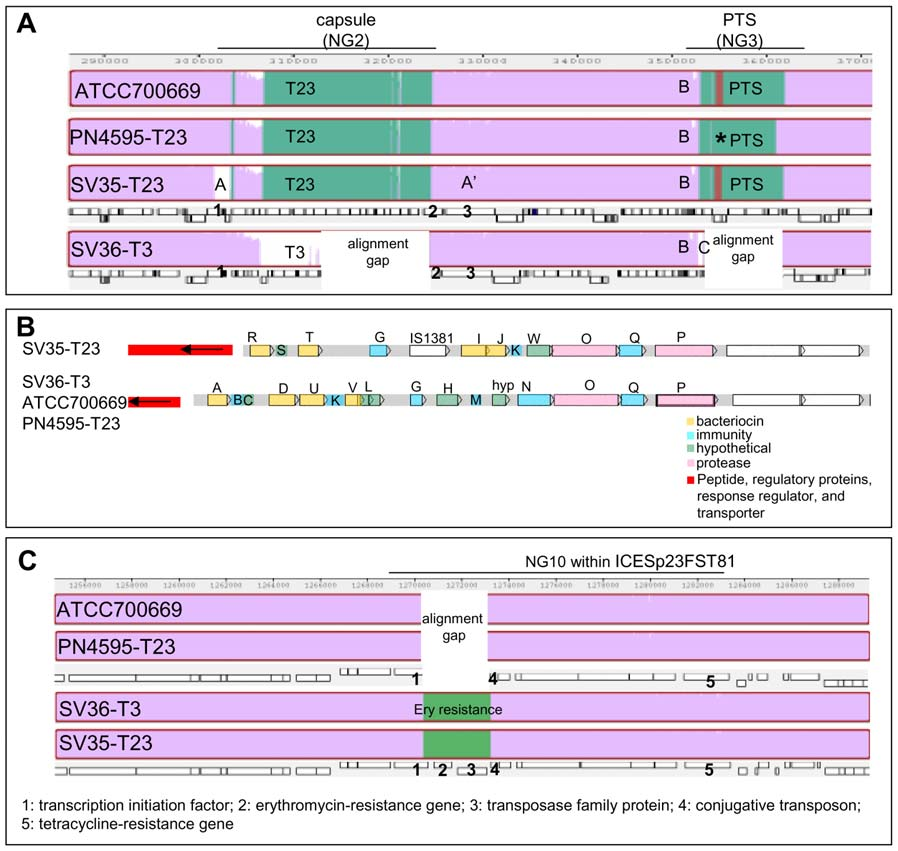
Detailed differences among PMEN1 strains. (**A**) Schematic of the capsule and flanking regions based on Mauve alignment of the four PMEN1 *S. pneumoniae* isolates. Purple denotes regions conserved amongst all strains; green highlights polymorphic regions, and gaps are represented in labeled white boxes. Asterisks mark a contig break, suggesting that the fragment apparently missing in PN4595-T23 is the result of a sequencing gap. Boxes below ATCC700669 and SV36-T3 mark the location of coding sequences, where dexB (5′ end of the capsule) is marked with a “1”, and aliA (3′ end of the capsule) with a “2”, and a cell wall surface anchored protein downstream with a “3”. An “A” denotes a transposase; An “A′ ” a cell wall protein that is variable in SV35-T23; A “B” marks a 9 Kb region with 89 SNPs differentiating SV35-T23 and SV36-T3 from each other or from the ATCC700669/4595-T23 pair; A “C” highlights the position of an integrase unique to SV36-T3. PTS: phosphotransferase system. (**B**) Variability within the bacteriocin (blp) loci of SV35-T23 versus the SV36-T3/4595-T23/ATCC700669 triplet. Position of all the coding sequences, highlighting genes for bacteriocins (yellow), immunity modulation (blue), proteases (pink), unknown function (green), and upstream two component signaling and transport systems (red.). (**C**) Schematic of the erythromycin resistance region based on MAUVE alignment of the four PMEN1 *S. pneumoniae* isolates. Purple denotes regions conserved amongst all strains; green highlights polymorphic regions, and gaps are represented in labeled white boxes. Boxes below ATCC700669 and SV36-T3 mark the location of coding sequences, where a “1” corresponds with a putative transcription initiation factor that is smaller in ATCC700669 and PN4595-T23, a “2” corresponds with the erythromycin-resistance gene, a “3” with a gene encoding a transposase family protein, a “4” with a conjugative transposon, and a “5” with a tetracycline-resistance gene.

As expected, SV36-T3 codes for a type 3 capsule while the other three strains contain genes which encode a type 23F capsule. In this region, SV36-T3 differs from the other strains by 855 SNPs and 21 genic differences (16 genes are exclusive to the type 23F strains, and 5 genes to the type 3 strain) (NG2 in Table 4). The region in ATCC700669 that contains the type 23 capsule and SNPs relative to SV35-T23 is 32617 bp long (Table 4), and includes 7542 bp upstream of Wze and 9311 bp downstream of RmlD. The area in SV36-T3 that contains the type 3 capsule and SNPs relative to the type 23 strains is 21039 bp long (8330 bp before the transposase and 7196 bp after the hypothetical protein at the end of the capsule). The genes present at these potential breakpoints are an upstream S-ribosylhomocysteinase and a downstream penicillin-binding protein 1A. These SNPs mark potential sites for recombination breakpoints. There are also differences among the serotype 23 loci, specifically the ATCC700669/4595-T23 pair and the SV35-T23 strain. These variations consist of differences in the transposase located at the 5′ end of the capsular operon and 20 SNPs on the cell wall anchored protein at the 3′ end of the capsule (marked by A and A′ respectively in Fig. 4A).

Previous work has shown that pneumococcal strains can undergo phase variation leading to transparent or opaque colony morphologies, where the opaque variants have increased systemic virulence potential [37]. The three type 23F strains yielded colonies of predominantly opaque appearance on agar plates, thus suggesting that the observed differences in virulence among the serotype 23 strains are not a consequence of phase variation. A 13.2 Kb region located 20 Kb downstream of the capsule also differentiates SV36-T3 from the type 23F strains (Fig. 4A). In this region, whereas the three type 23F strains contain six CDS, including a mannitol-specific phosphotransferase system (PTS), the SV36-T3 contains only an integrase gene (marked with a C in Fig. 4A); suggesting these genes were lost by SV36-T3 by an excision event. It is noteworthy that there is a 9 Kb region upstream of NG3 (marked with a B in Fig. 4A) where 89 SNPs differentiate ATCC700669/PN4595-T23 from either SV35-T23 or SV36-T3 or both. The presence of three diverse sequences in the regions surrounding the capsule (capsule borders and downstream region B) is consistent with multiple HGT events in this region leading to the observed differentiation. Accordingly, our RDP recombination analysis indicated that the region of SV36-T3 corresponding with NG3 was derived from an *S. pneumoniae* strain resembling strain AP200 (p = 6×10^−265^) [7], which is type 11A not type 3 further supporting separate events for the capsule switch and the PTS recombination event.

#### blp bacteriocin locus

A 42 Kb region, NG7 included ∼750 SNPs and 13 genes possession differences, differentiates SV35-T23 from the other three strains (Table 4). Ten of these distributed genes are present in the blp bacteriocin loci of the ATCC700669/PN4595-T23/SV36-T3 isolates and three are unique to the blp locus of SV35-T23 (Table S4). This locus encodes a two-component regulatory system that controls the expression of bacteriocins and immunity peptides shown to be critical in intra-species competition [35]. The locus may also play a role in systemic virulence, as demonstrated in the mouse pneumonia model where a deletion of the blp locus response regulator leads to a decrease in systemic virulence relative to the wild-type strain [38]. The regulatory portion of the locus consists of genes encoding a small peptide pheromone (blpC), a histidine kinase (blpH) and response regulator (blpR), and an ABC transporter (blpA-B) which cleaves and exports the pheromone, and the bacteriocins and their associated immunity proteins [39]. The four strains compared here have a TIGR4 like blpC sequence, but strain SV35-T23 differs from the other three strains by allelic differences in blpH, as well as genic differences in the bacteriocins and immunity peptides regulated by this two-component system. The origin of the variability of this locus in the PMEN1 strains is unclear, given that similar regions are widespread in the pneumococcus species. RDP analysis predicts that either the ATCC700669/PN4595-T23/SV36-T3 region was derived from a CDC3059-06-like strain, or that SV35-T23's region was derived from a D39-like strain. Similar regions have been described in detail in other strains, the bacteriocins and immunity proteins and blpH allele present in SV35-T23 are almost identical to that in the penicillin-sensitive strain 2306 [40], but 2306 has a different blpC allele. In contrast, the other three isolates have the same bacteriocins and immunity proteins as the PMEN1 strain F4 [40] (Fig. 4B).

The best-characterized intra-species bacteriocins are the neighboring blpM/N (also known as PncI/J, and labeled I and J in yellow in Fig. 4B), which are present in SV35-T23 but absent in the other three strains examined. Two allelic types of these peptides have been characterized for inhibitory activity against selected strains; they differ in six residues of blpM (PncI) and two residues of blpN (PncJ) [35], and only one of these alleles showed inhibitory activity against the other tested strains. SV35-T23 contains 7/8 of the polymorphisms present in the non-inhibitory allele set (CGSSpSV35_0665 and 0666, respectively). Strains SV35-T23, SV36-T3, and ATCC700669 were compared for their capacity to inhibit the growth of the highly sensitive indicator strain, P537 (strain 6A [41] with a deletion in the blp locus) [42]. In contrast to the strain 6A used as a positive control for growth inhibition, none of these PMEN1 strains showed evidence of inhibitory activity (data not show).

With the exception of the capsule (which has already been tested for its role in PMEN1 virulence (Hu et al. manuscript in progress 2011) the blp locus contains the largest number of genic differences between the phenotypically different strains, SV35-T23 and SV36-T3. Thus, we investigated whether the differences in bacteriocins and immunity proteins play a role in the pathogenesis of SV36-T3. To this end, we created a replacement mutant of SV35-T23 where the region of the blp locus encoding the bacteriocins and immunity proteins was replaced with that from SV36-T3, creating a SV35-T23SV36blp strain. The virulence phenotype of this mutant strain was compared to that of the parental SV35-T23 using the chinchilla OM model. Like the parental strain, infection with SV35-T23SV36blp showed very low mortality (1 out of 9 animals), little or no systemic disease (with the exception of the 1 animal that died, no other animals had systemic scores above 1), and comparable otologic disease (scores of 2 for 6 of the 9 animals) (compare to SV35-T23 in Fig. 1). The lack of a statistically significant difference between disease caused by infection with SV35-T23SV36blp or SV35-T23 demonstrated that the bacteriocins and immunity proteins in SV36-T3 are not sufficient to create a virulent strain in this model.

The operons surrounding the blp locus contain over 400 SNPs and a transposase that differentiates SV35-T23 from the remaining strains. This distribution is consistent with this region being acquired by SV35-T23 in a single horizontal gene transfer event that spanned the entire 42 Kb (RDP p-value 1×10^−308^).

#### Erythromycin Resistance Gene

The differences within NG10 are part of a previously characterized autonomous integrative and conjugative element (ICE), Tn916-like [8], [10]. This ICE is present in multiple strains, and in a subset of these strains it is nested within another Tn5252-like ICE element. In ATCC700669 this composite element is known as ICESp23FST81 and contains a tetracycline resistance gene (TetM) [8]. The analogous ICE in CGSP14 does not contain TetM but instead contains three genes that confer resistance to erythromycin (ermB), streptothricin (sat), and kanamycin (aph-3) [10].

The SV35-T23/SV36-T3 pair differs from the PN4595-T23/ATCC700669 pair by a 2.8 Kb insertion containing the ermB methylase gene that confers erythromycin resistance, a transposase family protein, and a conserved conjugative transposon protein (labeled 2–4 in Fig. 4C). This region adds a high degree of resistance to erythromycin for the SV35-T23/SV36-T3 pair in comparison to the other two strains which are sensitive (Table S5). The variation between these genotypes starts at the C-terminal end of a helix-turn-helix family protein (SPN23F12970) with a replication initiation factor domain, such that the form in the ATCC700669/4595-T23 isolates is shorter and does not contain a transcriptional repressor domain which is present in the longer version (labeled 1 in Fig. 4C). The surrounding regions also differ between these two strain pairs by 38 SNPs, 21 of which are located within the TetM gene (labeled 5 in Fig. 4C). The NG10 region, composed of the SNPs and genic differences in this area, is flanked by a putative conjugative transposon replication initiation factor on the 3′ end (SPN23F12970) and an integrase on the 5′ end (SPN23F13090), suggesting this region may have been acquired by a transposition event.

The entire 17 Kb of the NG10 region from the SV strains closely resembles the Hungary-19A-6 strain (99% identity over the entire region) and the CTn6002 element in *S. cristatus* (99% amino acid identity) [43]. The wide distribution of this region suggests it has been exchanged with pneumococcal strains outside of the PMEN1 lineage as well as among other streptococcal species.

#### MM1-2008 prophage

Fifty-one distributed genes make up the ∼40 Kb MM1-2008 prophage, which is absent in the SV35-T23/SV36-T3 strains and present in the other isolates (NG14). Two nearly identical variants of this prophage have been previously characterized, and the MM1-1998 variant has been implicated in improved pneumococcal adherence [44], [45]. The region surrounding this prophage differs between SV35-T23 and all the other strains by 11 SNPs upstream of the phage and 12 SNPs downstream of the phage, suggesting additional mutagenic events in this region.

#### Variable cell wall surface anchor proteins

Three of the NGs (NG1, NG13, and NG17) include differences in putative cell wall surface anchored proteins (CWSAP), and may reflect proteins that are under strong selective pressure by the host immune system. These proteins are annotated as CWSAPs by virtue of a N-terminal signal sequence and a C-terminal ‘LPxTG’ motif previously shown to anchor bacterial proteins to the cell wall [46].

The protein coded for at NG1 is an orthologue of the pneumococcal adherence and virulence factor B (PavB) and is different for each of the four genomes. This protein contains multiple streptococcal surface repeat protein domains (SSURE) that have been shown to bind fibronectin and plasminogen [47]–[49]. The PN4595-T23 and SV35-T23 forms of the gene are predicted to encode a 522aa protein, whereas the SV36-T3 gene is missing ∼150aa corresponding to one SSURE domain. The ATCC700669 orthologue has a variation on the N-terminus leading to a truncated N-terminal signal sequence which likely renders it a pseudogene.

The polymorphisms in NG13 span 13 genes (SPN23F14980-SPN23F15110), with the most significant differences occurring within a large CWASP gene predicted to encode a 1774aa protein (SPN23F_15110). This protein contains a G5 domain and three-collagen triple helix repeats (each one with at least 20 copies of the G-X-Y repeat that forms a triple helix), suggesting that it is likely associated with adhesion. Orthologues are found in many, but not all, of the *S. pneumoniae* strains, but differ significantly among these strains. However, the sequence of this gene is almost identical among the PMEN1 isolates with the exception being that SV35-T23 (CGSSpSV35_1653) contains an additional 52 residues including seven collagen repeats.

NG17 is contained within PsrP, a 4433aa coding sequence (SPN23F17820) that consists mostly of eight-residue repeats (sequence: TSASASAS) with an LPxTG containing C-terminus, but no signal peptide. This region shows variation amongst all four strains and RDP-analysis predicts three recombination events separately involving ATCC700669, PN4595-T23, and SV35-T23. However the low complexity of this large region complicates contig assembly and the polymorphism cannot be described with high certainty.

#### Restriction modification systems

The polymorphisms in NG6 are within a type I restriction modification protein S subunit adjacent to a phage integrase and lead to a highly variable coding sequence in SV36-T3. Similarly, the differences in NG8 are also within two type I restriction modification enzymes that surround a phage integrase and maintenance system. The sequence of the type I restriction modification enzyme in strains SV35-T23/SV36-T3 differs from that of strain ATCC700669 (the analogous region in PN4595-T23 is adjacent to a contig break and contains a stretch of poor quality sequencing data, thus this sequence has not been established with certainty). The middle domain of these enzymes is switched relative to the differing genotype; such DNA inversions between S subunits have been previously reported in bacteria and they are likely driven by selection for increased diversity within these enzymes [29], [50].

#### Transposases

A putative transposase differentiates SV36-T3 from the other strains at NG9, while a transposase which is predicted to be inactive differentiates SV35-T23 from the other strains at NG11.

### Comparison of ATCC700669 and PN4595-T23

The extremely high sequence similarity between ATCC700669 and PN4595-T23 (both serotype 23) is depicted in the schematic of Fig. 2. These strains have no differences in gene possession, and few allelic differences. Genome-wide these two strains differ at only 319 SNPs, of which only ∼220 have high quality sequencing scores. Of these 220 most probable SNPs, 139 are grouped in NG1, seven are within NG17, and the remainder are spread throughout the genome. Note that there are forty six gaps in the assembly of PN4595-T23 and, based on the difference in the predicted size for PN4595-T23 and ATCC700669, these gaps are predicted to contain ∼50 Kb of sequence (including ∼10 Kb around PsrP, ∼20 Kb distributed within many transposase/IS related CDS, ∼18 Kb within multiple regions dominated by rRNA, as well as multiple small gaps including RM systems and the MM1-2208 phage autolysin). Despite this high genotypic similarity, these strains exhibit statistically significant differences in systemic disease (Fig. 1C and Table 3). The mean for the maximal systemic score is 0 for ATCC700669 and 2.1 for PN4595-T23. This difference is significant as calculated by a t-test (p-value = 0.0058) and a non-parametric Wilcox test (p-value = 0.002). Forty percent of the animals infected with PN4595-T23 reached moribundity compared to none of the animals infected with ATCC700669 (Fisher-Exact p-value = 0.08). Moreover, *S. pneumoniae* was recovered from the brains of 80% of the animals infected with PN4595-T23, but none of the animals infected with ATCC70669 (Fisher-Exact p-value = 0.0007145).

The largest concentration of SNPs between these strains is in the PavB, already shown to play a role in pneumococcal virulence. Thus we tested whether the PavB polymorphism was sufficient to cause the pathogenicity difference between these strains. To this end we deleted the PavB gene from PN4595-T23 (strain PN4595-T23KOPavB) and replaced it with a spectinomycin-resistance cassette. This mutation did not significantly affect the systemic virulence of PN4595-T23 in the OM model, and suggests that the observed differences in the systemic virulence of PN4595-T23 and ATCC700669 cannot be solely attributable to the differences in their PavB genes.

## Discussion

Detailed comparison of the WGS from four *S. pneumoniae* isolates sharing common clonal type demonstrates phenotypic differences across strains, as well as more genomic variability within this lineage than has previously been revealed by other molecular typing methods. Strains ATCC700669, PN4595-T23, SV35-T23 and SV36-T3 display 94 gene possession differences and within their homologous sequences these strains differ from each other by ∼3500 SNPs. The great majority of these differences are concentrated within 18 neighboring groups (NGs) suggesting they may be the result of HGT events. In contrast to this variability, the high genomic similarity between strain ATCC700669 isolated in Spain in 1984, and PN4595-T23, isolated in Portugal in 1996, indicates that PMEN1 isolates can remain in the population for over a decade with only minor genotypic changes.

The genomic differences identified amongst the four strains studied here are consistent with those observed in a recent comparison of 240 PMEN1 strains [9]. The authors observed extensive recombination into the genomes of PMEN1 strains, and detected the highest frequency of HGT events in regions containing the capsule, drug-resistance genes, major surface antigens (such as pspA, psrP, and pspC), and prophage MM1-2008. In accordance, we also observed a capsule switch (to type 3), gain of erythromycin resistance (into strains SV35-T23 and SV36-T3 by changes in ICESpain23FST81), mutations in multiple CWAP including PsrP, as well as PavB and a putative collagen-like surface anchored protein, and differences in the presence of MM1-2008.

The combination of variable pathogenicity phenotypes and high genomic similarity make the PMEN1 set ideal for the identification of virulence determinants. Strains SV35-T23 and SV36-T3 have very different phenotypes in both the murine peritoneal infection model and the chinchilla OM model [19] (Hu manuscript in process 2011). A significant part of the virulence of SV36-T3 can be attributed to the type 3 capsule, since if the type 23 capsule in SV35-T23 is replaced by a type 3 capsule (creating SV35-T3) the recombinant strain is significantly more virulent (both otoscopically and systemically) than the SV35-T23 parent. Interestingly, however, in the otitis model the SV35-T3 is also less virulent than the SV36-T3 capsular donor [30] (Hu manuscript in process 2011). This SV35-T3 intermediate phenotype relative to the parental strains, suggests the type 3 capsule, while a major contributor to virulence, is not sufficient to account for the phenotypic difference between SV35-T23 and SV36-T3, and implicates additional pathogenecity gene(s) in strain SV36-T3. In the present study, we investigated the role of the bacteriocins and immunity peptides from the blp locus in the virulence of SV36-T3 by constructing strain SV35-T23SV36blp, a recombinant SV35-T23 strain with the bacteriocins and immunity peptides from SV36-T3. This region was selected because, with the exception of the capsule, it contains the largest number of genic differences between these two strains and has been previously implicated in virulence [38]. The lack of any statistically significant difference between the pathogenicity of the parental SV35-T23 strains and the SV35-T23SV36blp recombinant, suggests that the bacteriocins and immunity modulating proteins are not sufficient to account for the virulence in the chinchilla OM model. It is also possible that allelic differences in the blp regulatory proteins play a role in virulence. The gene encoding the response regulator, *blpR*, has already been implicated in virulence, since its deletion in a type 3 strain resulted in an attenuated phenotype in the mouse pneumonia model [38]. Furthermore, the blpC pheromone is sensed by the *blpH* gene product. Thus, it is possible that the allelic differences in *blpH* between SV36-T3 and SV35-T23 affect the capacity of these strains to respond to the TIGR4-like blpC, ultimately leading to variation in the immunity and bactericidal response of these strains *in vivo* [39].

Strain PN4595-T23 displays an intermediate systemic virulence relative to ATCC700669 and SV36-T3. This was surprising given that ATCC700669 and PN4595-T23 have a highly similar genomic content. The most notable difference between this pair is within the PavB coding sequence. Comparison between wild type and PavB deletion mutants in strain D39 have demonstrated that this adhesin plays a role in colonization of the respiratory airways. In the pneumococcal intranasal model, mice infected with the mutant strain showed an increased survival time and delayed spread of bacteria to the lungs. In the nasopharyngeal carriage model a reduction in carriage was observed for the mutant strains, and in a co-infection experiment the wildtype out-competed the mutant strain [49]. Given these data, chinchillas were inoculated with a recombinant PN4595-T23 where the PavB gene was deleted, to investigate its role in virulence. The absence of any significant difference in virulence between PN4595-T23 and the PavB deletion mutant in the OM model suggests that other regions may play a role in the pathogenecity of PN4595-T23, however, it is also possible that a difference in pathogenicity would be observed using a nasal, as opposed to bullar, inoculation route in the chinchilla. It is noteworthy that a two-component system (TCS08) implicated in cellobiose metabolism [51] and virulence [38] is located immediately downstream of the PavB gene. While there are no allelic differences in TCS08 between ATCC700669 and PN4595-T23 the possibility of polar effects from the differences in the PavB sequence cannot be excluded.

WGS of 44 *S. pneumoniae* strains has shown that independent isolates tend to be highly diverse. In fact, analyses of WGS of 44 clinical isolates have revealed that of all the genes that have been collectively detected in *S. pneumoniae* genomes (i.e. the *S. pneumonia* supra- or pan-genome) less than 50% were shared by all the isolates [11]. *S. pneumoniae* is naturally competent and variability in the gene complements of different strains is attributable to high rates of HGT. A particularly striking example for HGT in the clinical setting was described recently: strains isolated during the course of a single chronic (polyclonal) pediatric *S. pneumoniae* infection were shown to undergo more than 20 transformation events that accounted for the exchange of greater than 7% of the genome of the predominant strain [29]. In spite of this diversity, some clades displaying genomic diversity within them have been recovered on multiple continents over the last three decades. The PMEN1, PMEN2 (Spain^6B^ ST90) and PMEN3 (Spain^9V^ ST156), and the ST180 serotype 3 strains, represent such examples [11], [52], [53]. It is unknown whether the recent origin of this lineage, genic and/or physical isolation, or strong purifying selection favoring genetic strains has yielded the high degrees of genomic stability seen in these clades. Independent of the mechanism, PMEN1 strains have spread worldwide, and represent a high percentage of the drug-resistant strains such that understanding their variability and virulence is of critical importance to both specific diagnoses and treatment.

## Supporting Information

Figure S1 RDP3 Recombination analysis of four PMEN1 genomes and 7 additional S. pneumoniae strains, showing 11 recombinant regions. The 7 additional strains are AP200, CDC0288-04, CDC3059-06, D39, TIGR4, JJA, and CGSSp6BS73 and were selected to represent a genomically diverse group (with variable serotype and MLST types, see Material and Methods). The four green bars represent the chromosome of each one of the PMEN1 strains and the intervening gray boxes represent areas of recombination. In the cases where one of the 11 strains was identified as a likely donor (based on sequence similarity in the predicted recombinant region), the gray areas are labeled with the name of the likely DNA donor strain, if no donor was identified they are labeled “unknown”. The numbers above the boxes show the corresponding NG number, to correlate both analysis methods.
(TIF)

Table S1 Map of the final sequencing contigs onto the final assembly.
(XLS)

Table S2 Primers used to design SV35-T3-SV36blp and 4595-T23KOPavB.
(XLS)

Table S3 Scoring system to quantify otologic and systemic disease in the *S. pneumoniae* chinchilla model of OM.
(XLS)

Table S4 List of the 96 distributed gene clusters vatiable amongst the PMEN1 strains.
(XLS)

Table S5 Antibiotic MIC for *S. pneumoniae* strains.
(XLS)

## References

[1] Sa-Leao R, Nunes S, Brito-Avo A, Alves CR, Carrico JA (2008). High rates of transmission of and colonization by Streptococcus pneumoniae and Haemophilus influenzae within a day care center revealed in a longitudinal study.. J Clin Microbiol.

[2] WHO, W. H. O. (2008). Streptococcus pneumoniae.. http://www.who.int/vaccine_research/diseases/ari/en/index5.html.

[3] Brueggemann AB, Griffiths DT, Meats E, Peto T, Crook DW (2003). Clonal relationships between invasive and carriage Streptococcus pneumoniae and serotype- and clone-specific differences in invasive disease potential.. J Infect Dis.

[4] Forbes ML, Horsey E, Hiller NL, Buchinsky FJ, Hayes JD (2008). Strain-specific virulence phenotypes of Streptococcus pneumoniae assessed using the Chinchilla laniger model of otitis media.. PLoS ONE.

[5] Sandgren A, Sjostrom K, Olsson-Liljequist B, Christensson B, Samuelsson A (2004). Effect of clonal and serotype-specific properties on the invasive capacity of Streptococcus pneumoniae.. J Infect Dis.

[6] Bentley SD, Aanensen DM, Mavroidi A, Saunders D, Rabbinowitsch E (2006). Genetic analysis of the capsular biosynthetic locus from all 90 pneumococcal serotypes.. PLoS Genet.

[7] Camilli R, Bonnal RJ, Del Grosso M, Iacono M, Corti G (2011). Complete genome sequence of a serotype 11A, ST62 Streptococcus pneumoniae invasive isolate.. BMC Microbiol.

[8] Croucher N J, Walker D, Romero P, Lennard N, Paterson GK (2009). Role of conjugative elements in the evolution of the multidrug-resistant pandemic clone Streptococcus pneumoniaeSpain23F ST81.. J Bacteriol.

[9] Croucher NJ, Harris SR, Fraser C, Quail MA, Burton J (2011). Rapid pneumococcal evolution in response to clinical interventions.. Science.

[10] Ding F, Tang P, Hsu MH, Cui P, Hu S (2009). Genome evolution driven by host adaptations results in a more virulent and antimicrobial-resistant Streptococcus pneumoniae serotype 14.. BMC Genomics.

[11] Donati C, Hiller NL, Tettelin H, Muzzi A, Croucher NJ (2010). Structure and dynamics of the pan-genome of Streptococcus pneumoniae and closely related species.. Genome Biol.

[12] Hiller NL, Janto B, Hogg JS, Boissy R, Yu S (2007). Comparative Genomic Analyses of Seventeen Streptococcus pneumoniae Strains: Insights into the Pneumococcal Supragenome.. J Bacteriol.

[13] Hoskins J, Alborn WE, Arnold J, Blaszczak LC, Burgett S (2001). Genome of the bacterium Streptococcus pneumoniae strain R6.. J Bacteriol.

[14] Lanie JA, Ng W-L, Kazmierczak K, Andrzejewski TM, Davidsen TM (2007). Genome Sequence of Avery's virulent Serotype 2 Strain D39 of *Streptococcus pneumoniae* and Comparison with That of Unencapsulated Laboratory Strain R6.. J Bacteriol.

[15] Tettelin H, Nelson KE, Paulsen IT, Eisen JA, Read TD (2001). Complete genome sequences of a virulent isolate of *Streptoccus pneumoniae.*. Science.

[16] Feil EJ, Smith JM, Enright MC, Spratt BG (2000). Estimating recombinational parameters in Streptococcus pneumoniae from multilocus sequence typing data.. Genetics.

[17] Hanage WP, Fraser C, Tang J, Connor TR, Corander J (2009). Hyper-recombination, diversity, and antibiotic resistance in pneumococcus.. Science.

[18] McGee L, McDougal L, Zhou J, Spratt BG, Tenover FC (2001). Nomenclature of major antimicrobial-resistant clones of Streptococcus pneumoniae defined by the pneumococcal molecular epidemiology network.. J Clin Microbiol.

[19] Nesin M, Ramirez M, Tomasz A (1998). Capsular transformation of a multidrug-resistant Streptococcus pneumoniae in vivo.. J Infect Dis.

[20] Pletz MW, McGee L, Jorgensen J, Beall B, Facklam RR (2004). Levofloxacin-resistant invasive Streptococcus pneumoniae in the United States: evidence for clonal spread and the impact of conjugate pneumococcal vaccine.. Antimicrob Agents Chemother.

[21] Reinert RR, Ringelstein A, van der Linden M, Cil MY, Al-Lahham A (2005). Molecular epidemiology of macrolide-resistant Streptococcus pneumoniae isolates in Europe.. J Clin Microbiol.

[22] De Lencastre H, Tomasz A (2002). From ecological reservoir to disease: the nasopharynx, day-care centres and drug-resistant clones of Streptococcus pneumoniae.. J Antimicrob Chemother.

[23] Sa-Leao R, Tomasz A, Sanches IS, Nunes S, Alves CR (2000). Genetic diversity and clonal patterns among antibiotic-susceptible and -resistant Streptococcus pneumoniae colonizing children: day care centers as autonomous epidemiological units.. J Clin Microbiol.

[24] Delcher AL, Kasif S, Fleischmann RD, Peterson J, White O (1999). Alignment of whole genomes.. Nucleic Acids Res.

[25] Rozen S, Skaletsky H (2000). Primer3 on the WWW for general users and for biologist programmers.. Methods Mol Biol.

[26] Ewing B, Green P (1998). Base-calling of automated sequencer traces using phred. II. Error probabilities.. Genome Res.

[27] Gordon D (2004). Viewing and Editing Assembled Sequences Using Consed..

[28] Hogg JS, Hu FZ, Janto B, Boissy R, Hayes J (2007). Characterization and modeling of the Haemophilus influenzae core and supragenomes based on the complete genomic sequences of Rd and 12 clinical nontypeable strains.. Genome Biol.

[29] Hiller NL, Ahmed A, Powell E, Martin DP, Eutsey R (2010). Generation of genic diversity among Streptococcus pneumoniae strains via horizontal gene transfer during a chronic polyclonal pediatric infection.. PLoS Pathog.

[30] Hall BG, Ehrlich GD, Hu FZ (2010). Pan-genome analysis provides much higher strain typing resolution than multi-locus sequence typing.. Microbiology.

[31] Felsenstein J (1989). PHYLIP – Phylogeny Inference Package (Version 3.2).. Cladistics.

[32] Darling AE, Treangen TJ, Messeguer X, Perna NT (2007). Analyzing patterns of microbial evolution using the mauve genome alignment system.. Methods Mol Biol.

[33] Martin DP, Lemey P, Lott M, Moulton V, Posada D (2010). RDP3: a flexible and fast computer program for analyzing recombination.. Bioinformatics.

[34] Martin DP, Williamson C, Posada D (2005). RDP2: recombination detection and analysis from sequence alignments.. Bioinformatics.

[35] Dawid S, Roche AM, Weiser JN (2007). The blp bacteriocins of Streptococcus pneumoniae mediate intraspecies competition both in vitro and in vivo.. Infect Immun.

[36] Buchinsky FJ, Forbes M, Hayes J, Hu FZ, Greenberg P (2007). Virulence phenotypes of low-passage clinical oisolates of nontypeable Haemophilus influenae assessed using the chinchilla laniger model of otitis media.. BMC Microbiol.

[37] Overweg K, Pericone CD, Verhoef GG, Weiser JN, Meiring HD (2000). Differential protein expression in phenotypic variants of Streptococcus pneumoniae.. Infect Immun.

[38] Throup JP, Koretke KK, Bryant AP, Ingraham KA, Chalker AF (2000). A genomic analysis of two-component signal transduction in Streptococcus pneumoniae.. Mol Microbiol.

[39] de Saizieu A, Gardes C, Flint N, Wagner C, Kamber M (2000). Microarray-based identification of a novel Streptococcus pneumoniae regulon controlled by an autoinduced peptide.. J Bacteriol.

[40] Lux T, Nuhn M, Hakenbeck R, Reichmann P (2007). Diversity of bacteriocins and activity spectrum in Streptococcus pneumoniae.. J Bacteriol.

[41] Kim J O, Weiser JN (1998). Association of intrastrain phase variation in quantity of capsular polysaccharide and teichoic acid with the virulence of Streptococcus pneumoniae.. J Infect Dis.

[42] Son MR, Shchepetov M, Adrian PV, Madhi SA, de Gouveia l (2011). Conserved Mutations in the Pneumococcal Bacteriocin Transporter Gene, blpA, Results in a Complex Population Consisting of Producers and Cheaters.. MBio.

[43] Warburton PJ, Palmer RM, Munson MA, Wade WG (2007). Demonstration of in vivo transfer of doxycycline resistance mediated by a novel transposon.. J Antimicrob Chemother.

[44] Loeffler JM, Fischetti VA (2006). Lysogeny of Streptococcus pneumoniae with MM1 phage: improved adherence and other phenotypic changes.. Infect Immun.

[45] Obregon V, Garcia JL, Garcia E, Lopez R, Garcia P (2003). Genome organization and molecular analysis of the temperate bacteriophage MM1 of Streptococcus pneumoniae.. J Bacteriol.

[46] Schneewind O, Mihaylova-Petkov D, Model P (1993). Cell wall sorting signals in surface proteins of gram-positive bacteria.. Embo J.

[47] Bumbaca D, Littlejohn JE, Nayakanti H, Rigden DJ, Galperin MY (2004). Sequence analysis and characterization of a novel fibronectin-binding repeat domain from the surface of Streptococcus pneumoniae.. Omics.

[48] Jedrzejas MJ (2007). Unveiling molecular mechanisms of bacterial surface proteins: Streptococcus pneumoniae as a model organism for structural studies.. Cell Mol Life Sci.

[49] Jensch I, Gamez G, Rothe M, Ebert S, Fulde M (2010). PavB is a surface-exposed adhesin of Streptococcus pneumoniae contributing to nasopharyngeal colonization and airways infections.. Mol Microbiol.

[50] Dybvig K, Sitaraman R, French CT (1998). A family of phase-variable restriction enzymes with differing specificities generated by high-frequency gene rearrangements.. Proc Natl Acad Sci U S A.

[51] McKessar SJ, Hakenbeck R (2007). The two-component regulatory system TCS08 is involved in cellobiose metabolism of Streptococcus pneumoniae R6.. J Bacteriol.

[52] Hermans PWM, Overweg K, Sluijter, Groot R (2000). Penicillin-resistant Streptococcus pneumoniae: an international molecular epidemiological study..

[53] Vilhelmsson SE, Tomasz A, Kristinsson KG (2000). Molecular evolution in a multidrug-resistant lineage of Streptococcus pneumoniae: emergence of strains belonging to the serotype 6B Icelandic clone that lost antibiotic resistance traits.. J Clin Microbiol.

