# Supplementary figures and images for: Differences in Genotype and Virulence among Four Multidrug-Resistant Streptococcus pneumoniae Isolates Belonging to the PMEN1 Clone
Source: PLoS One. 2011 Dec 19;6(12):e28850. doi: 10.1371/journal.pone.0028850 (PMC3242761; doi:10.1371/journal.pone.0028850)

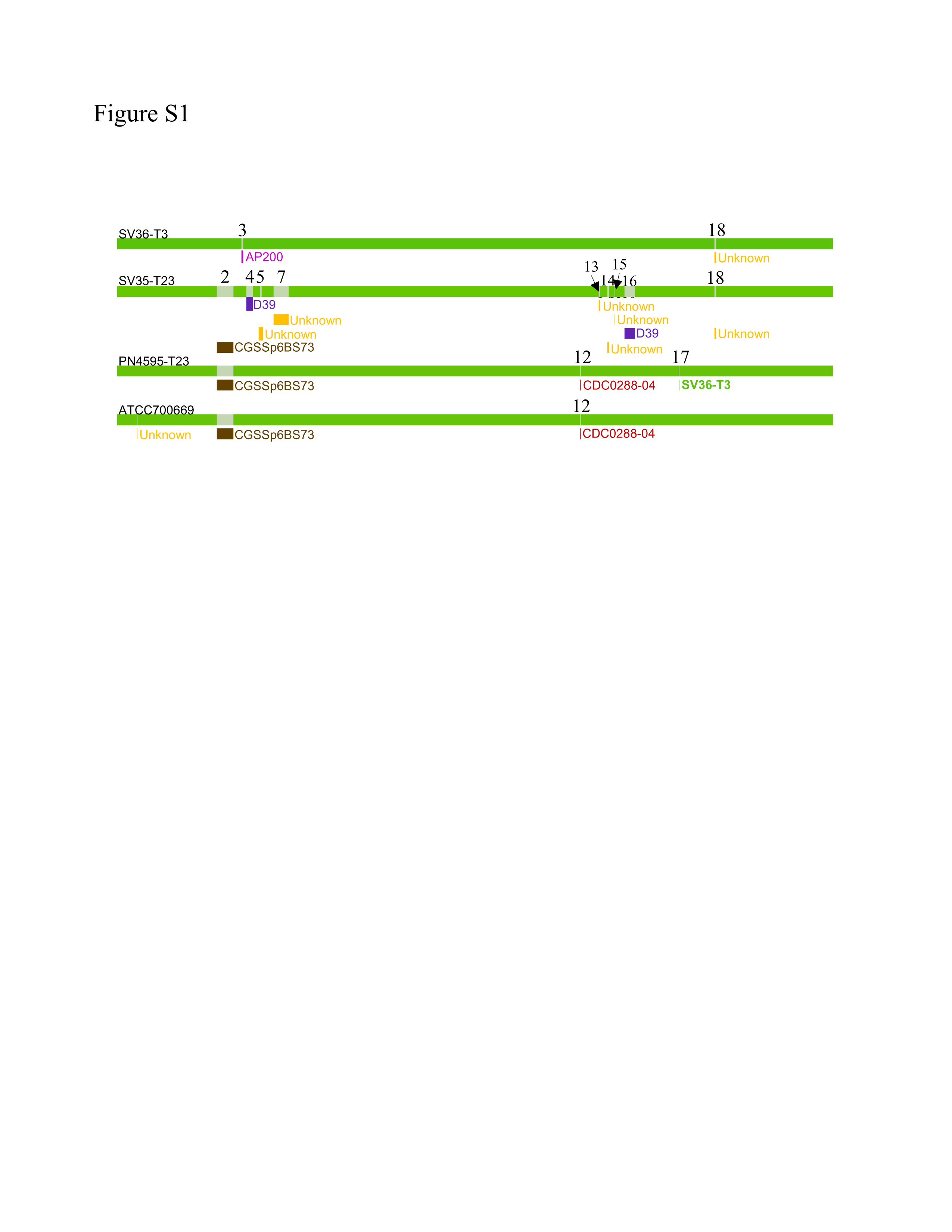

Supplement: Figure S1 — RDP3 Recombination analysis of four PMEN1 genomes and 7 additional S. pneumoniae strains, showing 11 recombinant regions. The 7 additional strains are AP200, CDC0288-04, CDC3059-06, D39, TIGR4, JJA, and CGSSp6BS73 and were selected to represent a genomically diverse group (with variable serotype and MLST types, see Material and Methods). The four green bars represent the chromosome of each one of the PMEN1 strains and the intervening gray boxes represent areas of recombination. In the cases where one of the 11 strains was identified as a likely donor (based on sequence similarity in the predicted recombinant region), the gray areas are labeled with the name of the likely DNA donor strain, if no donor was identified they are labeled “unknown”. The numbers above the boxes show the corresponding NG number, to correlate both analysis methods. (TIF) [file pone.0028850.s001.tif]
